# Implementation of an Interactive Voice Response System for Cancer Awareness in Uganda: Mixed Methods Study

**DOI:** 10.2196/22061

**Published:** 2021-01-26

**Authors:** Johnblack K Kabukye, Onaedo Ilozumba, Jacqueline E W Broerse, Nicolette de Keizer, Ronald Cornet

**Affiliations:** 1 Uganda Cancer Institute Kampala Uganda; 2 Department of Medical Informatics Amsterdam Public Health research institute Amsterdam UMC - Location AMC Amsterdam Netherlands; 3 Athena Institute Faculty of Science Vrije Universiteit Amsterdam Netherlands

**Keywords:** telemedicine, medical oncology, health promotion, low-and-middle-income countries, participatory research, mobile phone

## Abstract

**Background:**

Cancer awareness is crucial for cancer care and prevention. However, cancer awareness in Uganda is low, and access to cancer information is limited.

**Objective:**

This study aims to (1) understand the cancer awareness situation in Uganda (perceptions, beliefs, information needs, and challenges to accessing cancer information) and opinions about interactive voice response (IVR) systems; (2) develop cancer awareness messages and implement them in an IVR system; and (3) evaluate user acceptance and use of the IVR system.

**Methods:**

A participatory design approach was adopted. To understand cancer awareness needs and challenges, 3 interviews and 7 focus group discussions (FGDs) were conducted with cancer health care providers, patients with cancer, caregivers and survivors, administrators, and lay citizens (n=73). On the basis of the resulting qualitative data, audio messages addressing cancer information needs were developed and implemented in an IVR system. The system and messages were tested with users (n=12) during 2 co-design workshops before final rollout. Finally, the system was evaluated over 6 months after going live, using call records and user feedback from telephone interviews with callers (n=40).

**Results:**

The cancer information needs included general topics such as what cancer is, what causes it, cancer screening and diagnosis, cancer treatment, and practical information on what to expect during cancer care. There were also myths and misconceptions that need to be addressed, such as that cancer is due to witchcraft and has no treatment. Information on COVID-19 was also sought after following the outbreak. We developed 20 audio cancer messages (approximately 2 minutes each) in English and Luganda, along with 14 IVR navigation instructions. These were implemented in an IVR system with 24/7 availability from all over Uganda via a toll-free multi-channel telephone number. The total number of calls made to the IVR system 6 months after going live was 3820. Of these, 2437 (63.8%) lasted at least 30 seconds and were made from 1230 unique telephone numbers. There were 191 voice messages and 760 calls to live agents, most of which (681/951, 71.6%) were in Luganda. Call volumes peaked following advertisement of the system and lockdowns due to COVID-19. Participants were generally familiar with IVR technology, and caller feedback was largely positive. Cited benefits included convenience, toll-free access, and detailed information. Recommendations for improvement of the system included adding live agents and marketing of the system to target users.

**Conclusions:**

IVR technology provides an acceptable and accessible method for providing cancer information to patients and the general public in Uganda. However, a need remains for health system reforms to provide additional cancer information sources and improve cancer care services in general.

## Introduction

### Background

Cancer awareness is crucial for effective and satisfactory delivery of cancer care, and it is an important component of cancer control and prevention [1]. Cancer awareness refers to the knowledge and beliefs about the warning signs or symptoms of cancer, important risk factors, when to seek medical advice following each warning sign (urgency or seriousness of signs and symptoms) and cancer burden (knowledge of common cancers) [2,3]. It also encompasses knowledge and beliefs about cancer outcomes (eg, stigma and cancer fatalism [4,5]), available cancer services (eg, screening programs and recommended screening schedules and groups), and help-seeking intentions and perception of barriers (eg, worrying about wasting the physician’s time, the cost, or the distance to screening services) [2,3]. Low cancer awareness results in poor participation in cancer preventive measures (such as vaccination, smoking cessation, and screening), late presentation and diagnosis delay, nonadherence to treatment, poor coping, and overall dissatisfaction with cancer care [5-9].

Low- and middle-income countries (LMICs) bear a disproportionately large share of the global cancer burden [10,11], and low cancer awareness is often cited as a key contributing factor [7,8]. In Africa, general cancer awareness has been reported to be <40%, and awareness of cancer screening tests among at-risk populations is reported to be <20% according to Morhason-Bello et al [7]. A study on prostate cancer in Uganda [12] found that only 10.3% of respondents had good knowledge of the symptoms. Low awareness, negative beliefs, and myths (eg, belief that cancer is caused by witchcraft) have also been reported for breast [13] and cervical cancer [14] in Uganda, contributing to low screening rates (4.8% to 30%) and late presentation (over 80% of patients presenting with advanced disease) [15].

Mobile health (mHealth), defined as the use of mobile devices such as cell phones to support health [16], is potentially a cost-effective and acceptable tool for addressing low cancer awareness. There is growing evidence on the use and benefits of mHealth in different areas of healthcare in LMICs, particularly for facilitating communication, health education, and awareness of chronic illnesses [17-19]. mHealth can help overcome some of the greatest healthcare challenges in LMICs, such as geographical access (distance to healthcare facilities) and cost. Access to mobile phones is ubiquitous (over 90%), even in LMICs, and mobile phones are accepted across all demographic and socioeconomic groups [19,20].

However, there are several challenges that could hinder the success of mHealth interventions [17,19,21,22]. Problems with user acceptance are commonly reported due to users’ lack of familiarity with the technology, lack of cultural appropriateness or incentives to adopt new tools, and poor usability [17,21,22]. In LMICs, low literacy and infrastructural issues, such as reliable electricity and internet access, are also barriers [17,19,21]. Finally, the impact of health education and awareness interventions might be limited if they are not informed by theoretical underpinnings, which can help explain or predict behavior change following such interventions [23-27] or patient activation and engagement with the intervention [28-30].

In this paper, we describe an mHealth intervention to address low cancer awareness in Uganda. The intervention is an interactive voice response (IVR) system for dissemination of cancer information via telephone calls. In IVR, calls are automatically answered by a computer that plays back audio messages and navigation instructions. The caller interacts with the computer through voice commands or dual-tone multi-frequency signaling (DTMF) [31-35]. Our cancer information IVR system is based on qualitative research with key stakeholders and embodies established theories including the unified theory of acceptance and use of technology (UTAUT) [36], patient activation and engagement [28,37], and health belief model (HBM) [38,39]. IVR systems, which are commonly used by businesses such as telecoms and banks for customer relations, have not been used in cancer awareness or other health care interventions in general in Uganda.

### Aims

This study aims to (1) understand the cancer awareness situation in Uganda (perceptions, beliefs, information needs, and challenges to accessing cancer information) and opinions about IVR, (2) develop cancer awareness messages and implement them in an IVR system, and (3) evaluate user acceptance and use of the IVR system.

## Methods

### Approach

**Figure 1:**
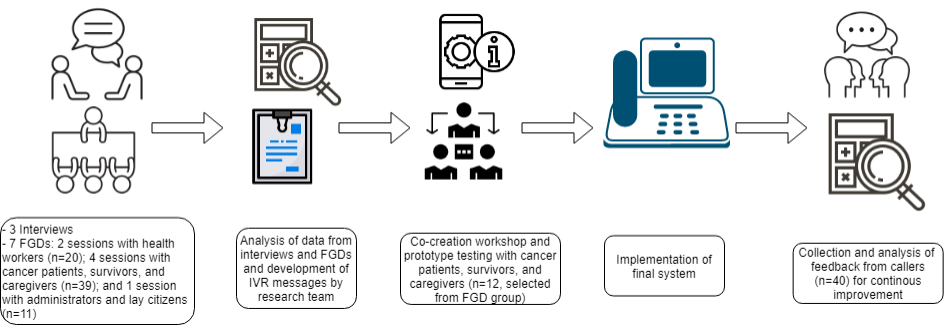
Summary of the approach to interactive voice response system development. FGD: focus group discussion; IVR: interactive voice response.

The study followed a participatory design approach [40] to develop the IVR system. In the participatory design methodology, users are involved in iterative phases of identification and analysis of user needs, prototype system development, testing and refinement, and summative evaluation [40-42]. This ensures a thorough understanding of user needs and contextual issues that might affect implementation and long-term adoption and also increases user empowerment and buy-in. To achieve this, we engaged participants in 3 forums: (1) qualitative key informant interviews and focus group discussions (FGDs), (2) co-creation workshops, and (3) user feedback through telephone interviews. We also quantitatively analyzed IVR system use by using call record details. Figure 1 summarizes this process.

### Setting

The project was implemented at the Uganda Cancer Institute (UCI) in Kampala, central Uganda. The UCI is the only comprehensive public cancer hospital in Uganda, serving over 5000 new cancer patients annually from Uganda and neighboring countries. The UCI runs a daily cancer screening and awareness clinic and conducts regular community outreach programs. The UCI also developed cancer education booklets that are given to clients who visit the institute or during outreach programs [43]. The interviews and FGDs were conducted in July 2019; co-creation workshops were conducted in October 2019, whereas the collection of system use data and feedback from callers (telephone interviews) was done in the first 6 months after going live (December 2019 to June 2020).

### Participants and Sampling Design

For the interviews, FGDs, and workshops, participants were purposively selected to represent different stakeholders, that is, cancer health workers who are responsible for providing cancer information and raising awareness (group 1); patients with cancer, survivors, or caregivers who would be the direct consumers of the cancer messages in the IVR (group 2); and other stakeholders, including administrators or policy makers and lay citizens (group 3). This diversity in participants was used to ensure data saturation [44,45].

For group 1, 2 research assistants (a patient counselor and a social worker with training in research ethics) approached patients and caregivers at UCI in person or called survivors who work with UCI in cancer awareness and advocacy.

For group 2, the first author (who is a medical doctor at UCI but was not directly involved in patient care at the time of the study) approached health workers at UCI in person to invite them for the interviews and FGDs. Different cadres of staff were invited, including physicians, nurses, specialist oncologists, pharmacists, palliative care specialists, and social workers.

For group 3, the first author invited administrators from UCI and policy makers from the Uganda Ministry of Health and Uganda Communications Commission, while the research assistants invited lay citizens.

FGD participants in group 2 were informed about the co-creation workshops that would follow, and those interested in participating provided contact information, which we used to invite them.

For the telephone interviews of callers, the participants included (1) those whom we had to call back after they had left voicemails on the system and (2) a selection of most and least frequent callers.

### Interviews and FGDs

We first conducted 3 key informant interviews with health workers (a palliative care physician, a cancer health educator, and a family physician in charge of cancer prevention) to obtain an overview of the cancer awareness landscape and develop the FGD guide. The interviews were unstructured, were conducted in English, and lasted about 1 hour each.

To stimulate discussion in the FGDs and to obtain rich data about a potentially sensitive topic, we used a vignette as the FGD guide [46]. The vignette represented the typical cancer journey of 2 characters, a man with prostate cancer and a woman with breast cancer. Cervical cancer, which is the most common cancer in Uganda, was avoided in the vignette, as the exploratory interviews revealed that an increasing knowledge of human papilloma virus as a sexually transmitted infection was leading to stigmatization of cervical cancer. The vignette is provided in Multimedia Appendix 1.

We conducted a total of 7 FGDs, each with 9 to 11 participants and lasting approximately 2 hours:

- 2 FGDs with group 1 participants (health workers), including both male and female participants depending on their availability. These were conducted in English.
- 4 FGDs with group 2 participants (patients, survivors, and caregivers) who were divided into relatively literate (FGDs in English) and relatively illiterate (FGD in Luganda, the most common local language), and further subdivided into male participants only and female participants only. Participants in this group were informed about the co-creation and system testing workshops that would follow, and those who would be interested were asked to provide consent and contact information.
- 1 FGD with group 3 participants (administrators, policy makers, and lay citizens), including both male and female participants. This was conducted in English.

In both the interviews and FGDs, we explored cancer awareness and information needs (topics) to be addressed by the IVR messages [2,3,5], including knowledge and beliefs about cancer signs and symptoms, causes or risk factors, stigma, perception of available cancer services, sources of cancer information, and challenges faced while accessing them. Specific attention was paid to constructs from HBM [38,39], including how participants perceived their susceptibility to cancer, the severity of the cancer problem, and benefits and barriers to cancer awareness. These guided the content and structure of the messages. Issues that could influence meaningful engagement with the intervention [28,37], for example, socioeconomic status, literacy, age, perception of the healthcare system, and self-efficacy with regard to IVR, were also noted. Finally, we explored participants’ attitudes and opinions about IVR using constructs from UTAUT [36], including ease of use and relative advantage (compared with other ways of getting cancer awareness information), and facilitating conditions or potential barriers. These informed the design choices for the IVR.

All sessions were moderated by the first and second authors and were audio-recorded and transcribed verbatim by research assistants who attended the session as note takers.

### Development of Cancer Messages and the IVR System

On the basis of the preliminary insights from the interviews and FGDs, we developed IVR content consisting of audio messages addressing the different cancer awareness topics as well as navigation instructions. The cancer messages were based on UCI’s cancer education booklets [43], and where lacking, these were supplemented by web-based material from the US National Cancer Institute [47] and our own clinical expertise. We deployed a prototype IVR system and invited participants from group 2 (patients, survivors, and caregivers) who had consented to be contacted to test and give feedback on the prototype in co-creation workshops. We held 2 workshops, one in English and the other in Luganda. Each had 6 participants (3 male and 3 female). We probed them about the clarity of information and instructions, flow of information or IVR menus (eg, *Treatment side effects* under the *Treatment* menu), voice preferences (eg, male voice vs female voice or voice of a familiar or famous person), etc. We worked with the participants to paraphrase the messages and rearrange them under the different IVR menus.

We then created the final system (described below), which went live in December 2019. We advertised it to patients in the patient waiting areas and encouraged them to share the toll-free number with their peers. The number is also printed on UCI patient appointment cards, and UCI staff were encouraged to tell patients about the IVR. The IVR was also advertised through a launch event that was covered by news agencies from central Uganda.

### Telephone Interviews of Callers and System Use Data

To evaluate the final system, we conducted 40 telephone interviews in which we asked the callers’ opinions about the IVR system. The telephone interviews were conducted by the first author and a research assistant (a nurse at UCI who is involved in cancer prevention and awareness) who also recorded the callers’ age, level of education, address (district in Uganda where the caller lives), mother tongue/dialect, reason for calling, and how the caller got to know about the system.

Moreover, the system automatically keeps a call detail record from which we obtained system use data such as the number of calls and IVR messages listened to and their time and date.

### Data Analysis

We qualitatively analyzed data from the interviews and FGDs by thematic analysis, as described by Braun and Clarke [48]. The first and second author, who had familiarized themselves with the content as they moderated the interviews and FGDs, independently read the transcripts, applied codes, and grouped similar codes into themes according to the theoretical constructs of HBM [38,39] and UTAUT [36]. We used the software package RQDA to assist in the qualitative analysis [49]. The themes were discussed between all the authors and with participants in the co-creation workshop to inform the design of the IVR system (menus and navigation instructions) and cancer messages. Multimedia Appendix 2 shows the themes, exemplary quotes, and the resulting system design decisions.

Quantitative data on system use (call records) were exported from the IVR system and cleaned to remove calls made to the system by the implementation team during testing. Of the remaining calls from clients, we excluded calls that were less than 30 seconds long, the amount of time it takes to listen to the first IVR instruction and make a selection off the IVR menu. This was to eliminate calls that could have been dropped or those where callers were just checking to confirm that the service is available but did not listen to the information. Analysis was performed in SPSS version 27 (IBM Corporation) using descriptive statistics.

### Ethics

The study was approved by the UCI Research Ethics Committee (UCIREC# 08-2019) and was registered by the Uganda National Council for Science and Technology (UNCST# HS418ES). All participants provided written informed consent before taking part in interviews, FGDs, or workshops and were given UGX 50,000 (approximately US $13) as reimbursement for their time and transport, as per the UNCST guidelines.

## Results

### Participants

Table 1 summarizes the characteristics of participants who were involved in the interviews and FGDs, and Table 2 summarizes the characteristics of participants who took part in the telephone interview. In total, there were 113 participants and 73 participants took part in the interviews, FGDs, and co-creation workshops during needs assessment and system development. Participants in the workshops (2 sessions each with 6 participants, balanced by sex) were invited from FGD group 2 (patients with cancer, survivors, or caregivers). In total, 40 callers took part in the telephone interviews after the system went live. The overall average age was 37.5 years (SD 13), with equal percentages of male and female participants. Approximately half of all the participants (57/113, 50.4%; particularly the patients, caregivers, or survivors in the FGDs and the callers of the IVR system) had an education level of secondary school or less and were not fluent in English.

**Table 1:** Characteristics of interview and focus group discussion participants (n=73).

Characteristic			Value^a^
**Forum, n (%)**			
	Interview		3 (4)
	**FGDs^b^**		
		Group 1 (health workers)	20 (27)
		Group 2 (patients with cancer, survivors, or caregivers)	39 (53)
		Group 3 (administrators and lay citizens)	11 (15)
**Sex, n (%)**			
	Male		32 (44)
	Female		41 (56)
**Education level, n (%)**			
	Primary school or none		19 (26)
	Secondary school		10 (14)
	College diploma		16 (22)
	Bachelor’s degree		21 (29)
	Master’s degree or higher		7 (10)
Age (years), mean (SD)			37.1 (9.1)

^a^The sum of percentages may not add up to 100% due to rounding.

^b^FGD: focus group discussion.

**Table 2:** Characteristics of the telephone interview participants (N=40).

Characteristic		Value^a^
**Sex, n (%)**		
	Male	26 (65)
	Female	14 (35)
**Education level, n (%)**		
	Primary school or none	13 (32)
	Secondary school	15 (37)
	College diploma	7 (17)
	Bachelor’s degree	3 (7)
	Master’s degree or higher	2 (5)
**How did you hear about the IVR^b^ service or how did you get the toll-free number, n (%)**		
	UCI^c^ patient appointment card or visit to UCI	8 (20)
	Radio or television	21 (53)
	UCI website or Google search	3 (7)
	Word of mouth outside UCI (eg, from friend or church)	8 (20)
Age (years), mean (SD)		35.0 (14.6)

^a^The sum of percentages may not add up to 100% due to rounding.

^b^IVR: interactive voice response.

^c^UCI: Uganda Cancer Institute.

### Cancer Awareness Situation in Uganda (Beliefs, Perceptions, Information Needs, and Challenges) and Opinions About IVR

Multimedia Appendix 2 shows the themes (based on the theoretical constructs of HBM and UTAUT), exemplary quotes from the interviews and FGDs, and how they influenced the design of the messages and IVR system.

The growing cancer burden in Uganda was well appreciated by the participants in all groups. They opined that rural areas are the most affected and described health system barriers to cancer services, especially in rural areas, such as limited cancer specialists and diagnostics. In addition, all participant groups reported that cancer awareness is low, both among the general public as well as among health workers, especially those not working directly in cancer care. They reported that there is a lack of access to cancer information and that myths and stigma are common. The examples mentioned by participants include the myth that cancer is caused by witchcraft, that diagnostic biopsies lead to rapid progression of cancer, a general belief that cancer is incurable, stigmatization of cancer patients because cancer is considered a curse, or that it can be transmitted from one person to another. Participants, particularly the less literate patients and caregivers, admitted that cancer patients are often discouraged from going for formal cancer care by their peers or are misinformed and duped by traditional healers (witch doctors), which results in delays in getting the right care.

There were also negative beliefs and misconceptions, particularly among the non–healthcare provider participants, about the referral process and healthcare system in general and about cancer as an illness that is complex. Participants expressed dissatisfaction with the general healthcare system reporting limited and poor services, such as insufficient drug stocks, long waiting times, and expensive care. Moreover, specialized care (such as cancer treatment) is perceived to have poor outcomes; therefore, referral to specialized hospitals like the UCI causes fear. Often, this makes people refrain from seeking cancer care at all or abandon treatment (loss to follow-up).

Some of the information needs that were highlighted from the interviews and FGDs included information on what cancer is, cancer signs and symptoms, cancer screening, diagnosis process, and treatment, and where these services can be availed. In addition, practical information on what to expect during the cancer journey (eg, cost and duration) is necessary so that patients and families prepare better. The participants also expressed a need for comforting and counseling information or services.

With regard to IVR, participants were generally familiar with the IVR technology from their experiences, for example, with customer service centers of telecom companies. They were positive about the potential of the IVR system as an avenue for dissemination of cancer awareness information because many own phones, can access information anytime and from anywhere, and it is free (calls are billed on the recipient). The participants advised that the IVR messages should be in multiple local languages; it should be marketed (eg, through bulk SMS or mass media) to make people aware of the existence of the service; and, if possible, it should be proactive where calls are initiated by the system as opposed to waiting for them to call because they might not take initiative. In addition, the use of voices of celebrities and public figures was suggested as a way to attract people to the service.

It was also emphasized that cancer survivors’ testimonies are very motivating and restore hope in cancer patients, for which reasons they should be included in the IVR system, as one participant revealed. Another suggestion was to recruit and train a pool of survivors and make the IVR system route calls to the survivors’ cell phones so that the survivors answer some questions or share their experience with callers. However, this is currently not in the system.

### The IVR System and Cancer Awareness Messages

The final system was deployed using FreePBX (version 14), an open source graphical user interface that controls the Asterisk private branch exchange (PBX) server (Sangoma Technologies Corporation) [50]. We installed the PBX on an HPE ProLiant DL380 Gen10 server with the following specifications: Intel Xeon 4110 (8 core, 2.1 GHz) processor, 16 GB RAM, and 1.2 TB storage (The Hewlett-Packard Company) [51]. We configured this to a Matrix Voice-over-Internet-Protocol gateway (Matrix Telecom Solutions) [52], which converts call traffic from a local (Ugandan) Integrated Services Digital Network (ISDN) into Session Initiation Protocol traffic and routes it to the PBX and back. The ISDN traffic is brought into the UCI using an E1 primary rate interface providing 30 simultaneous channels (calls). The calls are billed on the call recipient, so they are toll-free to the callers irrespective of their telecom provider (ie, the call costs are paid by the UCI). User interaction is through DTMF only. Voice recognition was not used because Uganda has many local languages (and accents) that are under-resourced with regard to natural language processing, and thus, there are no readily available voice recognition libraries [53]. The service is available 24/7 and has an option for users to leave a voicemail, allowing them to ask any questions or give feedback.

**Figure 2:**
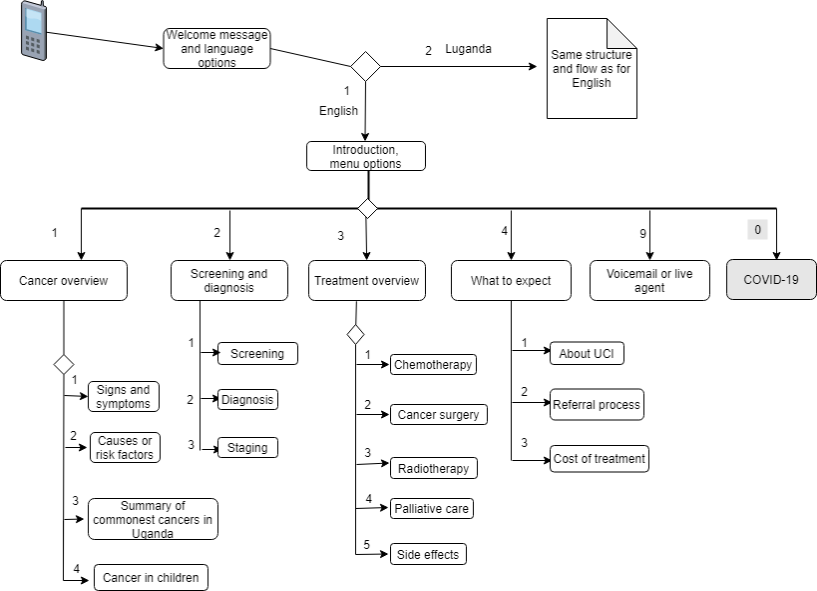
Interactive voice response menus and flow. The Luganda branch is not shown, but it is similar to the English branch. The numbers represent the dual-tone multi-frequency options that the caller has to enter to access the corresponding message. The options for COVID-19 information or to speak to a live agent were not part of the initial IVR system, but they were added 3 months after the system went live, as an emergency following the COVID-19 outbreak. At the end of each message, instructions are played for the caller to listen again, to go back to the main menu, or to go to voicemail or speak to an agent. IVR: interactive voice response; UCI: Uganda Cancer Institute.

The IVR menus were kept to a maximum of 5 options (with the exception of the COVID-19 option that was added as an emergency, see Figure 2), and the system was configured to allow sufficient time for a caller to enter their choice, repeating the instructions if input is missing or invalid. Testing of the system during the workshops showed that participants were able to navigate the IVR menus and easily find the information they were looking for. We only observed a usability issue with smart phones during the testing workshops, where after a call is placed, the keypad changes from the number dial pad to call control buttons. For those who were not used to this, it became impossible to enter numbers (DTMF) in response to the IVR menu options until a peer assisted them. We also observed some callers responding to the system by voice, for example, when asked to select language by pressing a number. There was no preference between male and female voices as long as the speech was not too fast and the accent was clear. For our system, we consistently used a female voice.

We developed a total of 20 voice messages, each approximately 2 minutes long, covering basic cancer topics such as what cancer is, its signs and symptoms, common cancers, risk factors, screening, and treatment. The messages also address the identified myths and misconceptions, offer hope and encouragement to patients and their families, and provide practical information on referral and care processes. Figure 2 shows the IVR menus and flow. We also developed 14 instruction messages for navigating the IVR menu. For example, the first interactions go as follows: “Thank you for calling the Uganda Cancer Institute, please choose your preferred language. For English, press 1, Bwooba oyagala kuwuliriza mu Luganda, nyiga 2 (Luganda for “If you want to listen in Luganda, press 2),” or later in the menu: “To learn what cancer is, press 1, to learn about cancer screening and diagnosis, press 2...”.

The navigation instructions and cancer messages are in 2 languages: English, which is the official language in Uganda, and Luganda, which is the commonest local language. We used simple terms, with unfamiliar medical terms and jargon used only when no simpler terms are available, and in that case, a detailed explanation is given. For example, a word that is often used for *Radiotherapy* in Luganda can be taken to mean *electrocution* or *roasting*. This can scare off patients, so we clearly explained this in the messages.

Following the corona virus outbreak and subsequent lockdown measures instituted in Uganda in March 2020, demand for health information via telephone increased. We, therefore, added COVID-19 information to the IVR system as well as an option for callers to speak to a clinician for individualized advice.

### Evaluation of System Acceptance and Use

At 6 months after going live (December 2019 to June 2020), a total of 3820 calls were made to the system. Multimedia Appendix 3 shows the distribution of calls across time of the day, day of the week, and over the 6 months.. We excluded 1383 (36.2%) calls that were less than 30 seconds long. The remaining 2437 (63.8%) calls were made from 1230 unique telephone numbers and lasted a total of 6646 minutes. On average, each telephone number called the system 3 times (SD 6.84; min=1, max=162, median 1). There were 191 voice messages with 143 (74.9%) in Luganda and others in English. Functionality to speak directly to a live agent (a member of the clinical team), as opposed to pre-recorded messages or voicemails, was added to the system on March 27, 2020, along with IVR messages on COVID-19. By June 5, 2020, 760 calls were made to live agents, with 538 (70.8%) in Luganda and the rest in English. The voice messages and calls to live agents were mostly to ask individualized questions that were not addressed by the IVR system. Call volume spiked immediately following advertisement of the service and following the lockdown due to the corona virus outbreak (Multimedia Appendix 3). As shown in Table 2, the information about the service spread out via different forums, including mass media and word of mouth.

The information that was most sought after is information explaining what cancer is, what causes it (risk factors), cancer screening and diagnosis, and practical information on what to expect during cancer care at the UCI (Multimedia Appendix 4). Information on COVID-19 was also frequently listened to, whereas information about cancer treatment was the least sought after.

Feedback from telephone surveys shows that callers appreciate the convenience of being able to access cancer information from wherever they are and at any time (including out-of-office hours) and the fact that it is toll-free, which removes the cost barrier. They also appreciate the detailed information provided by the system, which they could listen to over and again with no time limit. One of the most frequent callers said he called the system, although he was already physically present at the UCI for treatment, because the health workers are very busy and cannot spend much time explaining to him as the IVR system does.

A limitation of the IVR system that was noted from the feedback during the telephone interviews with callers is that the pre-recorded messages could not address some individualized or situational questions. Examples of these included specific symptom assessment (eg, “My mother has a lump in the breast” and “I have longstanding pain in the throat, could it be cancer?”), referral advice (eg, “I have a leg swelling with skin changes that is suspected to be cancer, which nearby health facility can I go to for cancer assessment?”), or how to live with cancer (eg, “my sexual function is affected by cancer treatment, what can I do?”). In addition, the messages provide a general overview of topics (treatment options, side effects, etc) that apply to all cancers or to the most common ones, yet some callers are looking for specific details such as cost of a specific radiology imaging test or information on a particular cancer, which might be less common and not covered by the system. We used the voicemail feature to partly solve these limitations, where callers could leave such individualized questions and we would call them back later to answer these questions. However, this approach was faced by several challenges, for example: (1) some callers shared phones, so when we called back, the person who answered denied leaving a particular voicemail question or even denied ever calling the system; (2) on some occasions, the caller could not be reached during call back or was busy and needed us to try again; and (3) some callers were unfamiliar with the voicemail feature, so they failed to leave messages because they did not hear a person speaking when the call went to voicemail. The addition of functionality to speak to a live agent solved this limitation and was applauded in the feedback surveys.

## Discussion

In this paper, we describe a participatory approach to the development, implementation, and initial experiences of an IVR for automating the dissemination of cancer information in Uganda. The ubiquity of mobile phones, familiarity with IVR technology, and positive attitude of target users mean that the IVR system is acceptable. The IVR system was perceived as advantageous in comparison with current alternative avenues for accessing cancer information, such as booklets or obtaining information from health workers. This is supported by the use patterns, where there are many returning callers, and feedback from callers is very positive. Callers were also able to obtain cancer information even during out-of-office hours such as weekends or at night (Multimedia Appendix 3) when health workers would not be accessible. The system has also become critically important for communication with patients during the corona virus outbreak, during which lockdowns make it difficult for patients to visit the hospital and obtain the necessary information.

Adoption of IVR overcomes many challenges facing cancer awareness efforts in Uganda, such as a limited number of health workers with expertise in cancer, low literacy, or limited access to the internet. The IVR system automates cancer education, requires limited reading literacy as voice is used, and works with basic telephone technology, including analog and second generation phones.

Previous studies have also found that IVR is acceptable as an avenue for accessing healthcare information in cancer care [33,54] and in other specialties such as child health [35,55] and mental health [56]. Similarly, considerations for increasing user acceptance and engagement with IVR systems, that is, using voices of celebrities or public figures, using survivors to provide peer support, and translation and contextualization of content, have also been reported [35,55,57,58] and are supported by theoretical constructs such as social influence from UTAUT [36]. Similarly, IVR system usability issues such as those with smartphone keypads have also been reported [35,55], and these need to be addressed to increase the ease of use, for example, through use of automated voice recognition.

A strength of this study is that we followed a participatory design approach to message and develop the IVR system and engaged key stakeholders in different forums (interviews, FGDs, co-creation workshops, and telephone surveys). This is important for gaining in-depth insights into the requirements and potential barriers to technology acceptance and use. In addition, we applied established theories (HBM and UTAUT) to inform the design and evaluation of our IVR system, which increases the reproducibility and effectiveness of the intervention [23-27].

A limitation of this study is that we did not evaluate the health outcomes, for example, change in cancer awareness or change in behavior (such as cancer screening rates or adherence to treatment) after implementation of the IVR system. Such evaluation of health outcomes is currently not feasible, as behavioral changes occur after a long time. Moreover, we implemented and tested one intervention for cancer awareness, that is, the IVR. Although this is supported by our findings from the interviews and FGDs, more studies, for example, randomized controlled trials comparing IVR versus other interventions for raising cancer awareness, would be needed to increase the strength of the evidence.

### Conclusions and Future Directions

Low cancer awareness is a recognized challenge to cancer control in Uganda, especially in rural areas. Key challenges facing access to cancer information include low literacy, limited number of cancer health workers who are limited to a few urban areas, and myths and misconceptions about cancer. A well-designed IVR system that is theory based and developed using participatory design approaches provides a convenient, accessible, and acceptable platform for the dissemination of cancer information that addresses the needs, as IVR is a familiar technology, mobile phones are ubiquitous, and the automation that comes with IVR reduces the burden on the health workforce. Moreover, mHealth solutions could be the only avenue for patients to access healthcare services under certain circumstances, as was the case with our system following disruptions due to COVID-19 lockdowns.

To improve the service of providing cancer awareness via phone, we recommend adding live call agents who have clinical knowledge about cancer care so that individualized questions can be answered in real time as opposed to voicemail. Marketing of the service is also important to ensure that potential users know about it and continue to use it. Technological solutions such as voice recognition and natural language processing could be used to allow callers to ask questions or navigate the IVR menus with voice commands, which will further improve usability.

Currently, we are in the process of translating the IVR system to add 5 additional Ugandan languages as well as adding more live agents. Future research will involve quantitative analysis of usability and task completion and evaluation of its health outcomes in comparison with other ways of cancer information provision.

## Appendix

Multimedia Appendix 1 Vignette used as the guide for the focus group discussions.

Multimedia Appendix 2 Coding of qualitative data into themes based on the constructs of the health belief model and unified theory of acceptance and use of technology.

Multimedia Appendix 3 Call trends across the time of day, day of the week, and 6-month period after system go-live.

Multimedia Appendix 4 Relative frequency (%) at which interactive voice response system messages are listened to. Top panel shows the options (topics), and bottom panel shows the subtopics.

## Abbreviations

DTMF: dual-tone multi-frequency
FGD: focus group discussion
HBM: health belief model
ISDN: Integrated Services Digital Network
IVR: interactive voice response
LMIC: low- and middle-income country
mHealth: mobile health
PBX: private branch exchange
UCI: Uganda Cancer Institute
UNCST: Uganda National Council for Science and Technology
UTAUT: unified theory of acceptance and use of technology

## References

[1] (2017). Guide to cancer early diagnosis. WHO.

[2] Simon AE, Forbes LJL, Boniface D, Warburton F, Brain KE, Dessaix A, Donnelly M, Haynes K, Hvidberg L, Lagerlund M, Petermann L, Tishelman C, Vedsted P, Vigmostad MN, Wardle J, Ramirez AJ, ICBP Module 2 Working Group‚ ICBP Programme BoardAcademic Reference Group (2012). An international measure of awareness and beliefs about cancer: development and testing of the ABC. BMJ Open.

[3] Stubbings S, Robb K, Waller J, Ramirez A, Austoker J, Macleod U, Hiom S, Wardle J (2009). Development of a measurement tool to assess public awareness of cancer. Br J Cancer.

[4] Vrinten C, Wardle J, Marlow LA (2016). Cancer fear and fatalism among ethnic minority women in the United Kingdom. Br J Cancer.

[5] Kobayashi LC, Smith SG (2016). Cancer fatalism, literacy, and cancer information seeking in the American public. Health Educ Behav.

[6] Niksic M, Rachet B, Duffy SW, Quaresma M, Møller H, Forbes LJ (2016). Is cancer survival associated with cancer symptom awareness and barriers to seeking medical help in England? An ecological study. Br J Cancer.

[7] Morhason-Bello IO, Odedina F, Rebbeck TR, Harford J, Dangou J, Denny L, Adewole IF (2013). Challenges and opportunities in cancer control in Africa: a perspective from the African Organisation for Research and Training in Cancer. Lancet Oncol.

[8] Sharma V, Kerr SH, Kawar Z, Kerr DJ (2011). Challenges of cancer control in developing countries: current status and future perspective. Future Oncol.

[9] Brédart A, Bouleuc C, Dolbeault S (2005). Doctor-patient communication and satisfaction with care in oncology. Curr Opin Oncol.

[10] Moten A, Schafer D, Farmer P, Kim J, Ferrari M (2014). Redefining global health priorities: improving cancer care in developing settings. J Glob Health.

[11] Bray F, Ferlay J, Soerjomataram I, Siegel RL, Torre LA, Jemal A (2018). Global cancer statistics 2018: GLOBOCAN estimates of incidence and mortality worldwide for 36 cancers in 185 countries. CA Cancer J Clin.

[12] Nakandi H, Kirabo M, Semugabo C, Kittengo A, Kitayimbwa P, Kalungi S, Maena J (2013). Knowledge, attitudes and practices of Ugandan men regarding prostate cancer. Afr J Urol.

[13] Scheel JR, Molina Y, Anderson BO, Patrick DL, Nakigudde G, Gralow JR, Lehman CD, Thompson B (2018). Breast cancer beliefs as potential targets for breast cancer awareness efforts to decrease late-stage presentation in Uganda. J Glob Oncol.

[14] Ndejjo R, Mukama T, Kiguli J, Musoke D (2017). Knowledge, facilitators and barriers to cervical cancer screening among women in Uganda: a qualitative study. BMJ Open.

[15] Nakisige C, Schwartz M, Ndira AO (2017). Cervical cancer screening and treatment in Uganda. Gynecol Oncol Rep.

[16] Iyengar S (2020). Chapter 12 - Mobile health (mHealth). Fundamentals of Telemedicine and Telehealth.

[17] Lewis T, Synowiec C, Lagomarsino G, Schweitzer J (2012). E-health in low- and middle-income countries: findings from the Center for Health Market Innovations. Bull World Health Organ.

[18] Hall CS, Fottrell E, Wilkinson S, Byass P (2014). Assessing the impact of mHealth interventions in low- and middle-income countries--what has been shown to work?. Glob Health Action.

[19] Hurt K, Walker RJ, Campbell JA, Egede LE (2016). mHealth interventions in low and middle-income countries: a systematic review. Glob J Health Sci.

[20] World B (2018). Information and communications for development 2018 : data-driven development. Open Knowledge Repository.

[21] Kruse C, Betancourt J, Ortiz S, Valdes Luna SM, Bamrah IK, Segovia N (2019). Barriers to the use of mobile health in improving health outcomes in developing countries: systematic review. J Med Internet Res.

[22] Gurupur VP, Wan TTH (2017). Challenges in implementing mHealth interventions: a technical perspective. Mhealth.

[23] Prestwich A, Sniehotta FF, Whittington C, Dombrowski SU, Rogers L, Michie S (2014). Does theory influence the effectiveness of health behavior interventions? Meta-analysis. Health Psychol.

[24] Webb TL, Joseph J, Yardley L, Michie S (2010). Using the internet to promote health behavior change: a systematic review and meta-analysis of the impact of theoretical basis, use of behavior change techniques, and mode of delivery on efficacy. J Med Internet Res.

[25] Prestwich A, Webb T, Conner M (2015). Using theory to develop and test interventions to promote changes in health behaviour: evidence, issues, and recommendations. Current Opinion in Psychology.

[26] Dalgetty R, Miller CB, Dombrowski SU (2019). Examining the theory-effectiveness hypothesis: a systematic review of systematic reviews. Br J Health Psychol.

[27] Avery KNL, Donovan JL, Horwood J, Lane JA (2013). Behavior theory for dietary interventions for cancer prevention: a systematic review of utilization and effectiveness in creating behavior change. Cancer Causes Control.

[28] Hibbard JH, Stockard J, Mahoney ER, Tusler M (2004). Development of the Patient Activation Measure (PAM): conceptualizing and measuring activation in patients and consumers. Health Serv Res.

[29] Graffigna G, Barello S (2018). Spotlight on the Patient Health Engagement model (PHE model): a psychosocial theory to understand people's meaningful engagement in their own health care. Patient Prefer Adherence.

[30] Schaeffer C (2017). Talk to Me. Oncology Issues.

[31] Tsoli S, Sutton S, Kassavou A (2018). Interactive voice response interventions targeting behaviour change: a systematic literature review with meta-analysis and meta-regression. BMJ Open.

[32] Kraft MR, Androwich I (2012). Interactive voice response technology: a tool for improving healthcare. NI 2012 (2012).

[33] Cohen-Cline H, Wernli KJ, Bradford SC, Boles-Hall M, Grossman DC (2014). Use of interactive voice response to improve colorectal cancer screening. Med Care.

[34] Franke KH, Krumkamp R, Mohammed A, Sarpong N, Owusu-Dabo E, Brinkel J, Fobil JN, Marinovic AB, Asihene P, Boots M, May J, Kreuels B (2018). A mobile phone based tool to identify symptoms of common childhood diseases in Ghana: development and evaluation of the integrated clinical algorithm in a cross-sectional study. BMC Med Inform Decis Mak.

[35] Brinkel J, Dako-Gyeke P, Krämer A, May J, Fobil JN (2017). An investigation of users' attitudes, requirements and willingness to use mobile phone-based interactive voice response systems for seeking healthcare in Ghana: a qualitative study. Public Health.

[36] Venkatesh V, Morris M, Davis G, Davis F (2003). User acceptance of information technology: toward a unified view. MIS Quarterly.

[37] Graffigna G, Barello S, Bonanomi A (2017). The role of Patient Health Engagement Model (PHE-model) in affecting patient activation and medication adherence: a structural equation model. PLoS One.

[38] Glanz K, Rimer B, Viswanath K (2008). Health behavior and health education: theory, research, and practice, 4th ed. Heal Behav Heal Educ Theory, Res Pract.

[39] Hou S (2014). Health education: theoretical concepts, effective strategies and core competencies. Health Promotion Practice.

[40] Clemensen J, Rothmann MJ, Smith AC, Caffery LJ, Danbjorg DB (2017). Participatory design methods in telemedicine research. J Telemed Telecare.

[41] Whittaker R, Merry S, Dorey E, Maddison R (2012). A development and evaluation process for mHealth interventions: examples from New Zealand. J Health Commun.

[42] Reeder B, Hills RA, Turner AM, Demiris G (2014). Participatory design of an integrated information system design to support public health nurses and nurse managers. Public Health Nurs.

[43] Information for cancer patients and the public. Cancer Information - Uganda Cancer Institute.

[44] Moser A, Korstjens I (2018). Series: practical guidance to qualitative research. Part 3: sampling, data collection and analysis. Eur J Gen Pract.

[45] (2018). The use of focus group discussion methodology: insights from two decades of application in conservation. Qualitative methods for eliciting judgements for decision making.

[46] Sampson H, Johannessen I (2019). Turning on the tap: the benefits of using ‘real-life’ vignettes in qualitative research interviews. Qualitative Research.

[47] Comprehensive cancer information. National Cancer Institute.

[48] Braun A (2006). Biosafety in handling gene transfer vectors. Curr Protoc Hum Genet.

[49] What is RQDA and what are its features?. RQDA Project.

[50] Freedom to communicate. freePBX.

[51] HPE ProLiant DL380 Gen10 Server - Overview. Hewlett Packard Enterprise Support Center.

[52] SETU VTEP. Fixed VoIP to T1/E1 PRI Gateway.

[53] Besacier L, Barnard E, Karpov A, Schultz T (2014). Automatic speech recognition for under-resourced languages: a survey. Speech Communication.

[54] Fadol AP, Mendoza TR, Lenihan DJ, Berry DL (2018). Addressing the symptom management gap in patients with cancer and heart failure using the interactive voice response system: a pilot study. J Adv Pract Oncol.

[55] Brinkel J, May J, Krumkamp R, Lamshöft M, Kreuels B, Owusu-Dabo E, Mohammed A, Bonacic Marinovic A, Dako-Gyeke P, Krämer A, Fobil JN (2017). Mobile phone-based interactive voice response as a tool for improving access to healthcare in remote areas in Ghana - an evaluation of user experiences. Trop Med Int Health.

[56] Janevic MR, Aruquipa Yujra AC, Marinec N, Aguilar J, Aikens JE, Tarrazona R, Piette JD (2016). Feasibility of an interactive voice response system for monitoring depressive symptoms in a lower-middle income Latin American country. Int J Ment Health Syst.

[57] Greaney ML, De Jesus M, Sprunck-Harrild KM, Tellez T, Bastani R, Battaglia TA, Michaelson JS, Emmons KM (2014). Designing audience-centered interactive voice response messages to promote cancer screenings among low-income Latinas. Prev Chronic Dis.

[58] Odeny TA, Newman M, Bukusi EA, McClelland RS, Cohen CR, Camlin CS (2014). Developing content for a mHealth intervention to promote postpartum retention in prevention of mother-to-child HIV transmission programs and early infant diagnosis of HIV: a qualitative study. PLoS One.

