# Supplementary material for: Implementation of an Interactive Voice Response System for Cancer Awareness in Uganda: Mixed Methods Study
Source: JMIR Mhealth Uhealth. 2021 Jan 26;9(1):e22061. doi: 10.2196/22061 (PMC7872833; doi:10.2196/22061)
Supplement: Multimedia Appendix 1 [file mhealth_v9i1e22061_app1.docx]

| **INTRODUCTIONS**  **Intro interview (objectives of the study)**  *Thank you for granting us time to discuss our planned intervention. We have requested this interview because we believe your knowledge will be instrumental to developing an intervention for people living with cancer. In this interview we will like to understand the needs of people living with cancer and their caregivers.. Do you have any questions for us?* |
| --- |
| **Intervention Description**  The goal of this study is to design an interactive voice recording system, known simply as IVR, for people living with Cancer in Uganda. IVR systems allow for the recording of selected information in appropriate languages and delivering them through mobile phones. The user can request the information via voice commands or by pressing a number on the phone keypad. Users can request this information at their own time including outside of the hospital and they can listen to messages as often as they would like. The information provided through this application will be tailored for patients at each phase of the cancer journey including detection, diagnosis, treatment, survivorship) |
| **Informed Consent**  You have been provided with an informed consent form to read. We will like to highlight some key information from it. All information you provide will be confidential, although results will be shared in reports and meetings, your personal information will be completely removed and your answers will not be associated with you. Your participation in this research is entirely voluntary. There are no consequences if you decide not to participate. You may also change your mind later and stop participating even if you agreed earlier. You will receive 20,000/- Uganda shillings as compensation for your time |
| **Do you have any questions?**  **Do we have your permission to record?** |

**Socio-demographics of participant**

Age:

Sex:

Highest Level of Education:

Profession:

Marital Status

*I would like to tell you a story about a man named Nsereko. Nsereko is 57 years old, he is married and lives in Luwero with his two wives and six children. He earns a comfortable living as a coffee grower. Nsereko has been generally healthy, except for occasional malarial episodes that gets treated at home. About 6 years ago he had surgery for a hernia. However, recently he has been experiencing worsening difficulty passing urine, and back pain that is has now limited his ability to move and work on his farm. Nsereko discusses with his first wife, Sanyu, about what may be wrong with him and what the next step. Sanyu, has heard some information about cancer and she thinks that maybe that is what Nsereko has.*

*What do you think happens next, please think for Nsereko as a man in your community and imagine what he would be feeling or thinking?*

- What does Nsereko think about his wife’s idea that this might be cancer
  - Does he believe her or not? Why or why not?
  - Where might Sanyu have gotten her information about cancer?
  - Who else might Nsereko discuss his current health situation with? Where else might he seek information?

*Nsereko visits different traditional healers and private clinics where he is given unspecified medication for pain, with only mild relief. Eventually Nsereko goes to a hospital to seek advanced care because his back pain is worsening and now has lost control of his urine. The doctor tells him it could be cancer, and several tests will be needed to confirm the diagnosis.*

- What do you think influences Nsereko decision to go to the hospital?
- What do you think he knows about the process of diagnosing cancer?
- Where will he have gotten his information?
- What do you think his experience of the diagnosis process will be?

*After 2 weeks of undergoing a series of tests including an ultrasound scan and a biopsy. Nsereko returns to his Doctors office and receives a diagnosis of prostate cancer. This news makes Nsereko very worried.*

- What worries might Nsereko have about being diagnosed with cancer?
- What might be his experience in the doctor's office? How is the information delivered? What questions might he have? How might the doctor respond to him?
- Who do you think might be the best person to give Nsereko the diagnosis news? Why?
- What conversation do you think Nsereko will have at home and within his community?
- How do you think the news about Nsereko’s diagnosis will be received by this community?

*After the diagnosis, Nsereke receives a referral to the UCI to begin treatment. We would like to discuss Nsereko’s next steps and related thoughts*

- What do you think Nsereko knows about possible treatment options? Where has he received this information? Do you think the information he has is accurate?
- Where can Nsereko seek more information about his diagnosis and treatment options?
- Do you think Nsereko returns to the hospital for treatment after his diagnosis? Why or why not?

*His first visit to the UCI is on a Monday morning and he shows up early with Sanyu for this visit. While sitting in the waiting area, Sanyu begins a conversation with Nabirye who is seated next to them. Nabirye has been receiving cancer treatment for about 2 months and is knowledgeable about the UCI hospital and cancer treatment process. Nsereko has many thoughts about what treatment could mean for him. Sanyu also has concerns for her husband but also her role as a caregiver. What questions do you think Nsereko and Sanyu will ask Nabirye?*

- What questions might Nsereko have about treatment and its effects?
- What could Nabriye tell Sanyu about the role of a cancer patient caregiver?
- What do you think does he expect from treatment? OR Why does he have these expectations of treatment?
- Might other people have different expectations?

*Now we will tell you a little bit more about Nabirye. Nabirye is 36 years old. She was born in Iganga and has been living in Kampala since she got married. She works as a bank teller while her husband is a construction. She is married and has four children. She was diagnosed with breast cancer 3 months ago and has been receiving treatment for the last 2 months.*

- Who do you think she would have discussed her diagnosis with?
- Who might she have consulted before making decisions about getting diagnosed and treated?
- What challenges might Nabirye encounter with receiving treatment?
- How do you think Nabirye’s cancer diagnosis and treatment affect her work? How does her work influence her cancer treatment process?
- How do you think the cancer diagnosis and treatment has affected or influenced Nabriye’s relationships with her husband, family and community?

*Over the next couple of months, Nsereko gets his treatment which includes chemotherapy and radiotherapy. Nabirye also completes her chemo, and gets a mastectomy. They become friends and after completing treatment they continue to attend the follow up clinic together. Although they are both very knowledgeable about the diagnosis and treatment phases of cancer , they have questions about what life after cancer treatment means for them.*

- What information might they want to know about their lives after cancer treatment?

- Where might they be able to get this information?

- What challenges might they face in their lives post cancer treatment?

Although Nsereko and Nabirye adhered to all treatment protocol there were many who began treatments with them and did not complete their treatment cycles. What might be some reasons for this?

*Nsereko and Nabirye remember a fellow patient, Namata, from Masaka, who had started treatment for cervical cancer around the same time as Nsereko but she never returned after her 3rd cycle of chemo.*

- What do you think could have led to Namata abandoning her treatment?
- What information do you think Namata needed to get so as to make an informed decision on whether to return for treatment or not?

*The doctors understand that Nsereko and Nabirye may not have received all the information they needed from their doctors visits. The hospital thinks that it will be a good idea to create a toll-free number which anyone seeking information for cancer can call to receive information on cancer, screening, diagnosis, treatment and support. When people call the toll free number they will receive a menu option to indicate what information they are calling about. For example the system may ask them to press 1 for information about cancer screening, 2 for cancer diagnosis, 3 for cancer treatment and 4 for cancer survivorship. Within each option will be additional information they can select options to receive additional details. We would like you to think about how such a system can work for answering the following questions.*

- How do you think Nsereko and Nabirye will feel about the option to call a toll free number?
- What do think would be the benefits of such a service?
- What kind of people do you think would benefit most from this service and why?
- What information do you think it should have?
- How long should it be?
- Do you have any idea of how information should be presented?
- What would be the challenges/difficulties associated with using this system?

*Thinking about your experiences with other systems such as the customer for banks or mobile networks. We would like to discuss what you thought about those systems*

- What worked about those systems? What did you like? What made it easy to use?
- What were your challenges with those systems? What did you not like? What made it hard to use?

*Thank you for all your attention and discussion with us today. We have come to the end of the discussion*

*We would like to ask if there is anything related to the topics we have discussed which we did not ask about but you would like to share with us?*

*Do you have any questions for us?*

*Thank you for your time!*
