# Supplementary material for: Implementation of an Interactive Voice Response System for Cancer Awareness in Uganda: Mixed Methods Study
Source: JMIR Mhealth Uhealth. 2021 Jan 26;9(1):e22061. doi: 10.2196/22061 (PMC7872833; doi:10.2196/22061)
Supplement: Multimedia Appendix 2 [file mhealth_v9i1e22061_app2.docx]

Coding of qualitative data into themes based on the constructs of health belief model and unified theory of acceptance and use of technology.

| Theme or theoretical construct | Description of finding | Quotes | Influence on IVR^a^ design and messages |
| --- | --- | --- | --- |
| Perceived severity of cancer in Uganda (HBM^b^: perceived severity) | All participant groups acknowledged that cancer is on the rise, yet there are few cancer specialists to provide cancer care and awareness. The few cancer care providers are limited to urban areas, so rural areas are disproportionately affected by lack of cancer services and low cancer awareness. As a result, many patients present with late stage cancer. Moreover, cancer fatalism was very commonly reported, especially among participants who are not health workers. Many thought that cancer has no treatment or is incurable, and that treatment is expensive and takes too much time, yet always ending in death. We also found that lack of understanding of the cancer care process and expected outcomes makes patients and caregivers frustrated with cancer care. Due to this, patients refrain from going to UCI^c^ (and instead prefer alternative healers) or abandon treatment | - “...people are suffering a lot with cancers in the villages there and they are unaware of it...cancer is a lot in the villages and we need a lot of sensitization to the people” (Participant 5 female patients/caregivers/survivors, English FGD^d^) - “Many patients present to the cancer institute when their cancer is grade 3 or 4 because they lack money to bring them to Kampala. The problem is the distance. Even if you want to seek medical intervention early, the doctors and nurses in the village clinics and health centers will say they do not have the gadgets to do the cancer tests, so they refer every suspect to Mulago [UCI] which is far for most of us.” (Participant 6, male, group 2, Luganda FGD) - “...initially, everyone thinks it is the usual things. I have a fever, or I have this, maybe it is malaria...we start from there...generally I think cancer does not have a specific symptom or sign until it is too late. That’s when people come to the Cancer Institute, when it is advanced.” (Participant 4, group 1, FGD) - “...a lady was referred for cancer management, so she thought on her coming she would do the tests in only one day, they prescribe her then she goes back or else if she’s to be admitted she will just be for two, three days then she goes back, but she left home a family. So when she came here they told her about biopsy that the results take roughly a week or two weeks you know all that and she’s getting weaker and weaker in the process of doing all these tests so the lady had to decide that ‘I can’t wait for this...I would rather go and take care of my child’…so they ended up going back, they withdraw from [treatment]... Her expectation was to finish the things fast and she goes back but only to reach here it’s roughly a month, she didn’t come with enough money and even if she is to call back home they won’t send the money...” (Participant 8, female, group 2, English FGD) | Justifies implementation of interventions to raise cancer awareness and these need to reach rural areas where there is greatest need. The messages cover a wide variety of cancer awareness topics including basic cancer biology, signs and symptoms, cancer screening and diagnosis, cancer treatment modalities and side effects, and practical information on cancer referral and care at UCI (Figure 2). We preemptively inform the caller that cancer care can be lengthy, but most often, it is received while a patient comes from home (they will not be admitted for the whole duration of care); explain the need for steps that are considered time consuming such as proper staging workup; and also explain the costs and other issues to expect when patients come to UCI |
| Susceptibility to cancer (HBM: perceived susceptibility) | Although participants agreed and showed concern about the increasing cancer incidence, there were sentiments and reports of denial and disbelief of a cancer diagnosis. This was due to lack of understanding of the causation or risk factors of cancer, with many considering it witchcraft. Particularly children were not thought to be susceptible to cancer, and this often results into marital conflicts when parents blame each other as responsible when a child gets cancer | - “...there are people who think they can never be affected or get cancer so such people may not really bother...” (Participant 3, female, group 2, Luganda FGD) - “...it took me some time to get to know that cancer is what was disturbing me. Because I was doing well with my petty-trade business as a woman, people told me that I had been witch-trapped and I believed. I used all the herbal medicine I got to know about. I consulted witch doctors but for a year, my leg continued to swell...It disturbed me a lot hearing the doctor say that it was cancer, I fainted. I stayed unconscious for about half a month. Many things were taking place including biopsy, but I was in my own world.” (Participant 4, female, group 2, Luganda FGD) - “I think that is because cancer is a strange disease and also because we lack faith. Like for me, when my child fell sick, some people said it was cancer, but I didn't believe.” (Participant 5, female, group 2, Luganda FGD) - “There is also the issue like who to blame it on. For instance in the children where people say: we don’t have cancer in the family and now the child has cancer, so you brought the cancer.” (Interview participant, cancer prevention) | The messages explain the risk factors for cancer, highlighting the common ones such as smoking and infections (Human Immunodeficiency Virus, Human papillomavirus, hepatitis, and *Helicobacter pyroli*). The messages also address the myths about witchcraft (message: “Cancer is not witchcraft or a curse”) and explain some causes that might not be very obvious such as genetic predisposition (*message:* “A person can develop cancer even when he or she does not have obvious risk factors”). We also added a section in the IVR menu specifically dedicated to cancer in children, explaining the unique characteristics |
| Barriers to cancer awareness (HBM: perceived barriers; refer to “Relative advantage of IVR” below in this table) | Several challenges to effective cancer control and creation of cancer awareness were identified, including: limited cancer experts and geographical barriers (cancer centers and experts are limited to urban areas), poor and costly cancer referral system, stigma, cancer fatalism, and other myths and misconceptions. There is also limited access to cancer information, and where information is available, it is in languages and formats that are difficult to consume (eg, limited reading literacy makes textual information inaccessible) | - “I did not know that there is cancer treatment center in Uganda until I fell sick. I first got to know about UCI when I went to Jinja. Where I come from, (Luka district) people are not aware of the services offered in Mulago.” (Participant 1, female, group 2, Luganda FGD) - “You have to be careful with the people you disclose to. For my case, when I disclosed to my father, he went on to tell people that I was to die any time. They started sharing everything I had worked for...You need to identify people who will be honest and look after your children, property, and other things. Because no one believed that I would return home alive, I found my house empty.” (Participant 6, female, group 2, Luganda FGD) - “...these caretakers...How will they make use of these booklets? They can't even read.” (Participant 7, female, group 3) - “I don't know about the others but me personally you get bored when you're reading something these articles are boring by the time we get to something that you want about that. You have to read causes, what, what so by the time you get to what you really want you are tired. So the audio is a very good thing” (Participant 5, female, group 3) - “There are many languages in our country,... there are about 65 ethnic groups...But there are few tribes that are very powerful whereby the language covers even other regions, one is that of Luganda, it can cover many other regions, almost half of the country, people will understand the language.” (Interview participant, health educator, male) | Justifies implementation of interventions to raise cancer awareness, and these need to reach rural areas where there is greatest need, and supports the choice of IVR as a mode of delivery for cancer awareness information since IVR technology is simple, familiar, and accessible. The messages are designed to give hope and reassure patients (message: “Cancer is treatable, and it can be cured if it is detected early and appropriate treatment is obtained”) and ask relatives and friends to be supportive of cancer patients rather than stigmatizing them. The first version of the system has information translated in 2 languages: English, which is the official language, and Luganda, which is the commonest local language. A follow-up phase of the implementation is ongoing, in which 5 additional local languages are being added |
| Cues to action (HBM) | Due to the reported lack of accurate information, it was apparent that many people in Uganda do not know what to do to prevent cancer risk factors or how and where to seek care when they are suspected to have cancer, which leads to delays. The participants also highlighted the need to market the system/service so that it is known to people. They also suggested proactive pushing of the messages to people if possible instead of waiting for them to call, although with care to privacy and avoiding causing undue fear | - “Yes, it is a long journey. You spend a lot, you get different advice, spend money with witch doctors because you think you are being bewitched. People advise you to go here and there” (Participant 7, female, group 2, Luganda FGD) - “I think the system needs to send text messages using the phone networks in different languages. After reading the message, someone picks interest to seek and read further. For example, if the message says to get more information press this number. Without the initial message, people will not know that such a system exists.” (Participant 3, female, group 2, Luganda FGD) - “...remember the 2016 elections, I don’t want to say whether I liked it or not, but I got a call with the president’s voice, asking for a vote and I tried to ask him something but he didn’t reply [referring to a push IVR system that was used in political campaigns]. So, by the end of the day, I got to know that someone was asking for a vote. It would be very good, to push. There are very many messages we are not reading, if I know it is from advertising, but at least for a call, most people will pick the call, and when they get that number.” (Participant 5, health worker, FGD) - “This is health information that is allowed to be pushed through but at the end of it all if you do not want to receive this information, dial this and you opt out. Also...they can get information that sometimes worsens their fears...at the end of it if I have fears I want at the end of getting this information to be encouraged to go to the hospital.” (Participant 7, female, group 3) | We added cues to action throughout all the messages, for example, message on signs and symptoms: “if you have [these] symptoms and signs and they last for long eg 2 to 4 weeks, or if they rapidly get worse, please see a qualified health worker to check if it is cancer or not, and to get the appropriate treatment”, or another message on treatment side effects: “you should always ask your doctor or nurse about the side effects of the treatment you are going to receive, and speak up about the side effects or any other problems that you might experience during cancer treatment.” The messages also explain the referral process and what to expect when patients go to the UCI, encouraging them to come for health education and check-up even when they do not have symptoms. We have also advertised the IVR number in the hospital and media; the number is printed on patient appointment cards, and we encourage the health workers at UCI to inform the patients about it |
| Ease of use of IVR (UTAUT^e^) | Generally, the IVR is familiar as it is routinely used in Uganda by customer care departments of telecoms and other service providers, and it was considered easy to use. Key considerations for ensuring better usability that participant emphasized include using simple terms when sending the messages, translating to different languages, and avoiding long waiting times before callers access information | - “...my only humble request is whoever is going to develop that make it user-friendly. Very simple for all… the starting point is the language you're going to use whether it is Luganda let it be the Luganda that someone can understand… whether it is in English let it be English which a primary school student can understand...the words or the way you explain should be, you know, clear to each and everybody.” (Participant 11, female, group 3) - “One of the problems I have encountered with telecom customer care is being put on hold. Sometimes you want immediate help, but the call gets picked and they put you on hold.” (Participant 1, male, group 2, Luganda FGD) - “...when you want to talk to let’s say customer care [of a teleco] they say hold on the line, may be somebody is busy they will get back to you, then you keep there for about ten minutes now for sure if I want to inquire something like faster faster will that person hold on?” (Participant 8, female, group 2, English FGD) - “I think it should also have a provision of speaking to the doctor because even for customer care in the service sector, there is a provision of speaking to a person if you want.” (Participant 1, male, group 2, Luganda FGD) | Supports the choice of IVR as a mode of delivery for cancer awareness information. The automation of the calls ensures callers do not have to wait for live agents to get information. System has multiple channels, allowing several concurrent calls, and there is a voicemail function to allow callers to leave a message instead of waiting for an agent. An option to speak directly to a health worker was also added. Messages are in plain languages as much as possible. When there are no simpler words to accurately substitute medical jargon (eg, biopsy and radiotherapy), these words are used, but they are explained clearly |
| Relative advantage of IVR (UTAUT) | IVR was considered more advantageous compared with alternative means of obtaining information about cancer, for example, cost and convenience | - “...you spend less time than having to travel to the place” (Participant 4, female, group 2, Luganda FGD) - “Information can reach the whole of Uganda in a short time. Instead of doctors traveling to many places to create awareness, people can call from anywhere in Uganda and get the information. If MTN and Airtel networks are used, they are everywhere in Uganda.” (Participant 1, female, group 2, Luganda FGD) - “It is even free. They do not charge any airtime” (Participant 3, female, group 2, Luganda FGD) - “You can call any time, even late in the night like 2 am” (Participant 1, female, group 2, Luganda FGD) | Supports the choice of IVR as a mode of delivery for cancer awareness information. System is available 24/7. The IVR is accessible using a toll-free number (callers are not billed, instead the UCI is billed for the calls) |
| Social influence (UTAUT) | Participants opined that using voices of public figures or celebrities could encourage more people to listen to the IVR messages and take the necessary actions. They also suggested using cancer survivors’ testimonies in the IVR or to recruit and train survivors to answer calls, especially when there are no health workers to answer calls. This is because the personal stories of cancer survivors are more inspiring and give hope | - “I did not have hope of living for another year because while I was still on the ward, many patients died but one time, when I had gone to radiotherapy for the first time, I sat next to a lady who was saying that she got radiotherapy in 1980. I could not believe it. Her statement gave me a lot of hope. It made me eager to get radiotherapy too” (Participant 7, female, group 2, Luganda FGD) - “Survivors who have gone through similar experiences should counsel the patients. Doctors and nurses give the treatment, but they do not have real life experiences like the survivors.” (Participant 3, female, group 2, Luganda FGD) | These suggestions are being considered for the next phases in which a full-fledged hospital call center system will be implemented |
| Contextual and demographic factors (HBM and UTAUT) | Mobile phones are ubiquitous in Uganda, and network coverage is countrywide. All demographic groups have access to phones | “...access to phone has gone higher...according to the UCC It is more than 75% access and almost every adult whether he is illiterate, is holding a phone. At least in every household there is one...If a woman is not having, the husband is having, if the husband is not having, their daughter is having.” (Interview participant, health educator, male) | Supports the choice of IVR and other mobile health solutions as a mode of delivery for cancer awareness information |

^a^IVR: interactive voice response.

^b^HBM: health belief model.

^c^UCI: Uganda Cancer Institute.

^d^FGD: and focus group discussion.

^e^UTAUT: unified theory of acceptance and use of technology.
