# Supplementary figures and images for: Implementation of an Interactive Voice Response System for Cancer Awareness in Uganda: Mixed Methods Study
Source: JMIR Mhealth Uhealth. 2021 Jan 26;9(1):e22061. doi: 10.2196/22061 (PMC7872833; doi:10.2196/22061)

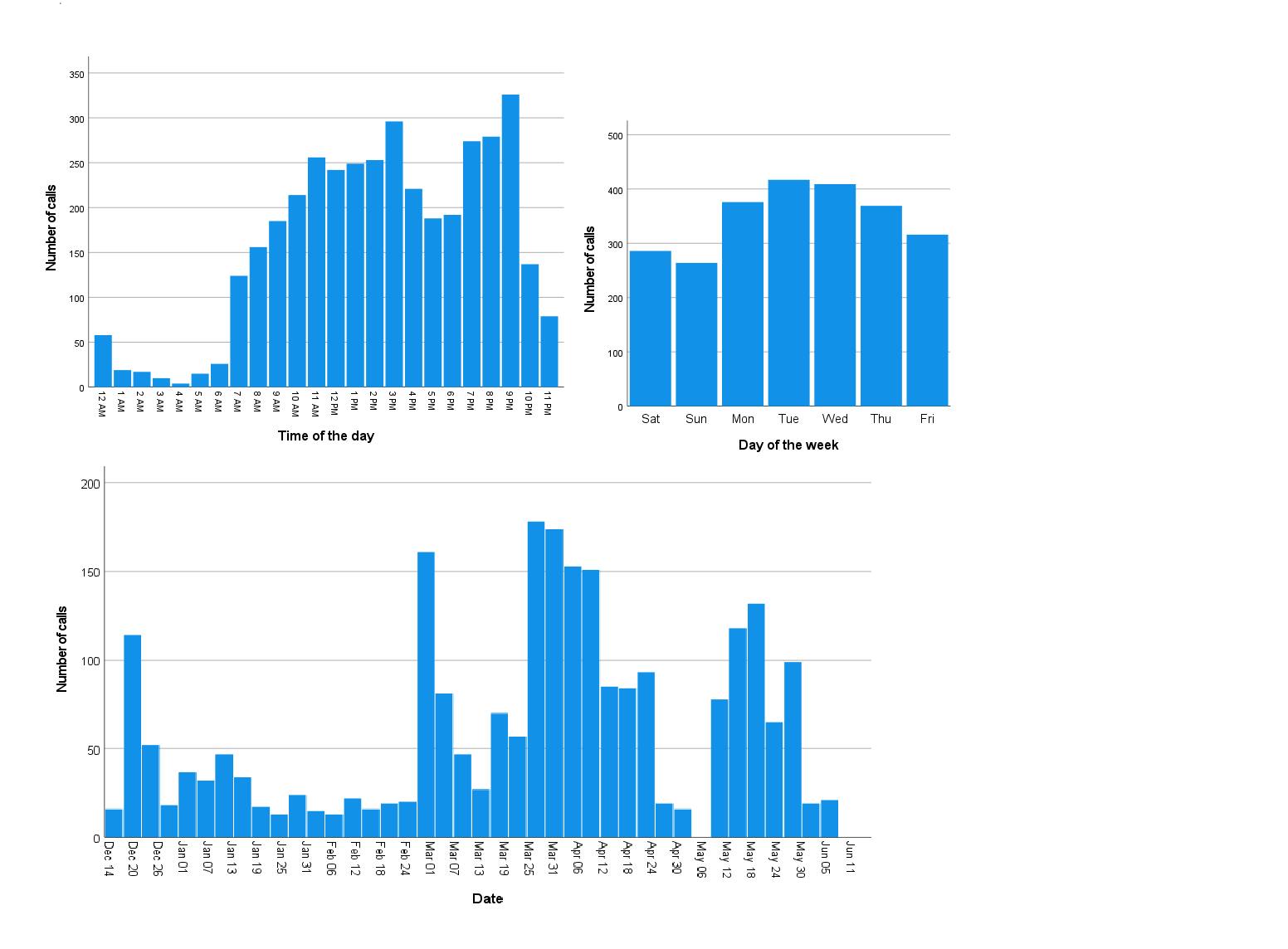

Supplement: Multimedia Appendix 3 [file mhealth_v9i1e22061_app3.png]

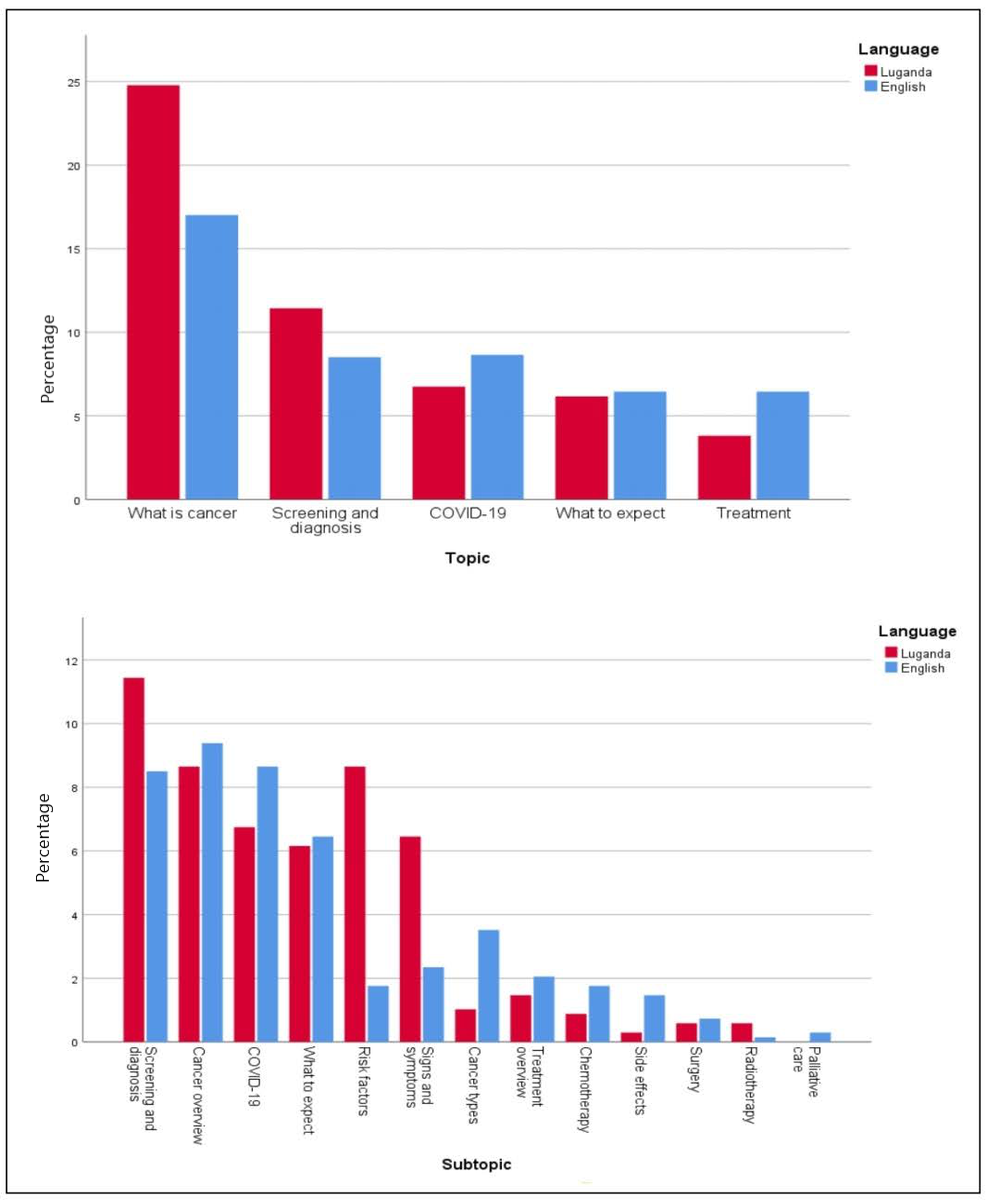

Supplement: Multimedia Appendix 4 [file mhealth_v9i1e22061_app4.png]
